# A cross‐sectional study of the radiation dose and image quality of X‐ray equipment used in IVR

**DOI:** 10.1120/jacmp.v17i4.6231

**Published:** 2016-07-08

**Authors:** Yohei Inaba, Koichi Chida, Ryota Kobayashi, Masayuki Zuguchi

**Affiliations:** ^1^ Department of Radiological Technology Graduate School of Health Sciences Tohoku University #1 Sendai Japan; ^2^ Department of Disaster Medical Science International Research Institute of Disaster Science Tohoku University #2 Sendai Japan

**Keywords:** interventional radiology (IVR), image quality, radiation dosimetry, fluoroscopy, cineradiography

## Abstract

There are case reports of injuries caused by the radiation from interventional radiology (IVR) X‐ray systems. Therefore, the management of radiation doses in IVR is important. However, no detailed report has evaluated image quality for a large number of IVR X‐ray systems. As a result, it is unclear whether the image quality of the X‐ray equipment currently used in IVR procedures is optimal. We compared the entrance surface doses and image quality of multiple IVR X‐ray systems. This study was conducted in 2014 at 13 medical facilities using 18 IVR X‐ray systems. We evaluated image quality and simultaneously measured the radiation dose. Entrance surface doses for fluoroscopy (duration, 1 min) and cineradiography (duration, 10 s) are measured using a 20‐cm‐thick acrylic plate and skin dose monitor. The image quality (such as spatial resolution and low‐contrast detectability) of both fluoroscopy and cineradiography was evaluated using a QC phantom. For fluoroscopy, the average entrance surface dose using the 20‐cm‐thick acrylic plate was 13.9 (range 2.1–28.2) mGy/min. For cineradiography, the average entrance surface dose was 24.6 (range 5.1–49.3) mGy/10 s. We found positive correlations between radiation doses and image quality scores, in general, especially for fluoroscopy. The differences in surface dose among the 18 IVR X‐ray systems were high (max/min, 9.7‐fold for cineradiography; 13.4‐fold for fluoroscopy). The differences in image quality scores (spatial resolution, low‐contrast detectability, and dynamic range) were also very large. In general, there tended to be a correlation between radiation dose and image quality. Periodical measurements of the radiation dose and image quality of the X‐ray equipment used for cineradiography and fluoroscopy in IVR are necessary. The need to minimize patient exposure requires that the dose be reduced to the minimum level that will generate an image with an acceptable degree of noise.

PACS number(s): 87.57.C, 87.57.uq, 87.59.B, 87.59.bf, 87.59.C, 87.59.cf, 87.59.Dj

## I. INTRODUCTION

Interventional radiology (IVR) confers a higher risk of radiation‐related injury to both the patient and physician compared with other types of X‐ray examination.^1^, ^2^, ^3^, ^4^, ^5^, ^6^, ^7^, ^8^, ^9^, ^10^, ^11^, ^12^ Even today, there are case reports of injuries (such as radiation‐induced erythema) caused by the radiation from IVR X‐ray systems.^13^, ^14^, ^15^ Therefore, the management of radiation doses in IVR is important.^16^, ^17^, ^18^, ^19^, ^20^, ^21^

The International Commission on Radiological Protection (ICRP) developed the concept of a “diagnostic reference level” (DRL).^(22)^ DRLs are used to indicate whether, under routine conditions, patient dosage levels are unusually high or low for a given procedure.^(22)^ The difficulty level for IVR procedures has a wide range, from relatively easy procedures to complex ones. In all probability there may be no such thing as a “routine condition” in IVR. In other words, actual IVR procedures will vary considerably depending on the complexities of the case. However, we believe that the DRL for IVR has significance. In fact, the ICRP has stated that each facility should include, in the clinical protocol for IVR procedures, a statement on dose rates and patient skin dose, and this statement in the protocol provides the IVR physician with baseline levels for patient skin dose.^(1)^

A few studies have evaluated radiation doses in several IVR X‐ray systems.^23^, ^24^, ^25^, ^26^ To our knowledge, however, no detailed report has evaluated image quality (such as spatial resolution and low‐contrast detectability) for a large number of IVR X‐ray systems. Therefore, it is unclear whether the image quality of the X‐ray equipment currently used in IVR procedures is optimal.

In this study, we compared the entrance surface doses and image quality of multiple IVR X‐ray systems, and investigated the optimization of the radiation dose and image performance in IVR X‐ray systems.

## II. MATERIALS AND METHODS

This study was conducted in 2014 at 13 medical facilities using 18 IVR X‐ray systems in and around Sendai, Japan (Table 1).

### A. Radiation dose measurement

The methods used for measuring the radiation dose have been described.^25^, ^26^ Briefly, entrance surface doses for fluoroscopy (duration, 1 min) and cineradiography (duration, 10 s) are measured using a 20‐cm‐thick acrylic plate and skin dose monitor (SDM, model 104–101, McMahon Medical Inc., San Diego, CA). The X‐ray conditions used for these fluoroscopy and cineradiography measurements in this study, including the dose mode, image receptor (flat‐panel detector (FPD) or image intensifier, (I.I.)), field magnification mode, and recording speed (pulse or frame rate), were the conditions normally used by the facilities performing percutaneous coronary intervention (PCI). The entrance exposure area was the area associated with the actual diameter setting of the image receptor (FPD or I.I.).

The entrance surface dose, corrected for the cine frame rate (i.e., the frame dose), was determined as the frame dose (mGy/frame)=[original entrance surface dose with cineradiography(mGy/s)/cine recording speed(frames/s)]. The entrance surface dose, corrected for the fluoroscopy pulse rate (i.e., the pulse dose), was also calculated as the pulse dose (mGy/pulse)=[original entrance surface dose with fluoroscopy(mGy/min)/fluoroscopy pulse rate(pulse/s)].

**Table 1:** The characteristics of our study

*Facility*	*1*		*2*	*3*	*4*	*5*			*6*		7		*8*	*9*	*10*	*11*	*12*	*13*
*X‐ray System*	*A Infinie Celeve* −i 8000V *TOSHIBA*	*B Infinti Celeve* −i 8000V *TOSHIBA*	*C Bransist Safire SHIMADZU*	*D Advantx GE*	*E Infinti Celeve DP TOSHIBA*	*F Axiom zee biplane SIEMENS*	*G Infinti Celeve* −i 8000V *TOSHIBA*	*H Infinti Celeve* −i 8000V *TOSHIBA*	*I Infinie Celeve* −i 8000C *TOSHIBA*	*J Infinie Celeve* −i 8000F *TOSHIBA*	*K Axiom zee biplane SIEMENS*	*L Axiom Artis dBC SIEMENS*	*M Trinias B‐12 SHIMADZU*	*N Axiom Artis BA SIEMENS*	*O Bransist Safire SHIMADZU*	*P Infinix Celeve* −i 8000V *TOSHIBA*	*Q Allura Xper FD20 PHILIPS*	*R Allura Xper FD10 PHILIPS*
Image receptor	FPD	FPD	FPD	I.I.	FPD	FPD	FPD	FPD	FPD	FPD	FPD	FPD	FPD	I.I.	FPD	FPD	FPD	FPD
Actual diameter [cm]	16.4	16.4	18.0	14.5	16.4	13.5	11.6	11.6	14.0	18.5	13.5	13.5	14.3	14.2	14.5	18.6	14.9	14.9
Years of use	2.6	2.6	4.2	12.0	8.7	1.2	7.6	2.0	0.5	6.8	1.5	7.8	0.5	10.0	5.5	7.0	5.0	10.0
Total filtration [mm]	2.5 Al	2.5 Al	1.5 Al	2.5 Al	2.8 Al	2.5 Al	2.5 Al	2.5 Al	2.5 Al	2.5 Al	2.5 Al	2.5 Al	1.5 Al	2.5 Al	1.5 Al	2.3 Al	2.5 Al	2.5 Al
Grid ratio [:1]	13	13	10	10	13	15	13	13	13	13	15	15	13	17	10	13	15	13
*Fluoroscopy*																		
Tube potential [kV]	80.0	80.0	77.0	80.0	80.0	90.8	70.0	70.0	70.0	70.0	68.4	70.0	76.0	77.0	73.0	80.0	91.0	78.0
Pulse rate [pulses/s]	10.0	10.0	12.5	Cont.	15.0	7.5	7.5	7.5	7.5	7.5	7.5	7.5	15.0	7.5	15.0	15.0	15.0	15.0
Additional filtration [mm]	0.3 Cu	0.3 Cu	1.5 Al+0.6 Cu	‐	0.06 Ta	0.9 Cu	0.2 Cu	0.2 Cu	0.2 Cu	0.2 Cu	0.2 Cu	0.2 Cu	1.5 Al+0.6 Cu	0.9 Cu	1.5 Al+0.3 Cu	0.06 Ta	1.0 Al+0.4 Cu	0.4 Cu
Entrance dose [mGy/min]	10.5	10.8	8.8	22.1	21.9	2.1	18.8	18.5	18.7	8.8	8.3	9.3	12.5	5.2	28.2	12.2	17.1	16.8
Pulse dose [mGy/pulse]	0.018	0.018	0.012	0.012	0.024	0.005	0.042	0.041	0.042	0.020	0.018	0.021	0.014	0.012	0.031	0.014	0.019	0.019
*Image quality score*																		
Spatial resolution	3.0	3.5	3.0	3.5	4.0	1.0	5.0	6.0	6.0	5.0	3.5	4.0	4.0	3.0	4.0	4.0	4.0	4.0
Low contrast detectability	2.0	2.0	1.5	1.5	2.0	0.0	4.0	4.0	4.5	2.5	2.0	3.0	2.0	2.5	4.0	2.0	2.0	2.0
Dynamic range	3.5	4.0	3.5	3.0	5.0	1.5	5.0	5.5	5.0	5.0	4.0	4.0	5.5	4.0	5.0	5.0	5.0	3.5
*Cineradiography*																		
Tube potential [kV]	74.0	73.0	68.0	80.0	74.0	76.0	74.0	73.0	74.0	74.0	72.4	68.3	70.0	76.2	68.0	71.0	68.0	71.0
Frame rate [frames/s]	15.0	15.0	12.5	12.5	15.0	15.0	15.0	15.0	15.0	15.0	10.0	10.0	15.0	15.0	15.0	15.0	15.0	15.0
Additional filtration [mm]	1.8 Al	1.8 Al	1.0 Al	‐	0.03 Ta	0.3 Cu	1.8 Al	1.8 Al	1.8 Al	1.8 Al	0.1 Cu	0.1 Cu	1.0 Al+0.01 Au	0.1 Cu	1.5 Al+0.01 Au	1.8 Al	1.0 Al+0.4 Cu	0.1 Cu
Entrance dose [mGy/lOs]	16.3	16.6	24.7	28.1	44.5	6.3	41.3	37.8	49.3	17.6	5.1	9.9	19.1	23.2	27.2	29.5	27.1	19.4
Frame dose [mGy/frame]	0.109	0.111	0.198	0.225	0.297	0.042	0.275	0.252	0.329	0.117	0.051	0.099	0.127	0.155	0.181	0.197	0.181	0.129
*Image quality score*																		
Spatial resolution	5.0	5.0	5.0	5.0	5.0	4.0	6.0	7.0	6.0	6.0	5.0	5.0	5.0	4.5	5.0	5.0	5.0	5.0
Low contrast detectabiliry	3.0	3.5	4.0	3.5	4.0	2.0	5.0	5.5	4.5	4.0	3.5	4.0	3.0	4.0	4.5	3.5	3.5	4.0
Dynamic range	5.0	6.5	7.0	3.5	7.0	4.0	10.5	10.0	7.0	7.5	4.5	4.5	7.0	6.0	7.5	6.5	6.5	6.5

FPD=flat‐panel detector; I.I.=image intensifier; Cu=copper; Al=aluminium; Ta=tantalum; Au=gold.

### B. Image quality evaluation

**Figure 1:**
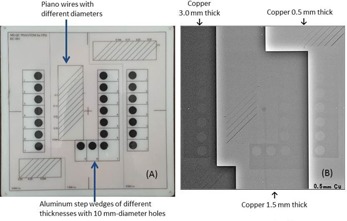
The QC phantom used in our study. Phantom size: $20*20({\text{cm}}^{2})$. The phantom comprises piano wires and aluminum step wedges placed on Cu bases with three thicknesses. (a) Appearance of the QC phantom, (b) X‐ray image of the QC phantom; around the center of the X‐ray image, the SDM sensor and cable are slightly visible.

The image quality of both fluoroscopy and cineradiography was evaluated using a QC phantom (KC‐001, Mitaya Manufacturing, Saitama, Japan; Fig. 1), consisting of three thicknesses of copper (0.5, 1.5, and 3.0 mm), an aluminum step‐wedge (0.1–2.7 mm thick, with 10‐mm‐diameter holes), and piano wires of various diameters (0.08–0.5 mm). The phantom facilitates visual evaluation of the spatial resolution, low‐contrast detectability, and dynamic range of the system.^(27)^

The measurement geometry used to evaluate the image quality was similar to that presented in the radiation dose measurement. The phantom was placed on an acrylic plate sandwiched by the image receptor. The X‐ray output (radiation dose) used for the evaluation was adjusted to the same level as the surface doses given above for each of the X‐ray systems.

### C. Spatial resolution

The minimum diameter of the piano wire on the 1.5‐mm‐thick copper base of the phantom was visually evaluated on a display monitor using the fluoroscopy and cineradiography images. The identifiable minimum diameter was scored from 1 to 6 using minimum respective visualized diameters of 0.5, 0.4, 0.35, 0.3, 0.25, and 0.2 mm (i.e., the score increased with the spatial resolution).

### D. Low‐contrast detectability

The minimum thickness of the aluminum step‐wedge (i.e., the minimum thickness of the aluminum in which a hole could be detected) on the 1.5‐mm‐thick copper base of the phantom was also evaluated visually, using the fluoroscopy and cineradiography images on an X‐ray display monitor, and scored from 1 to 6 using the minimum visualized thickness of 1.3, 1.1, 0.9, 0.7, 0.5, and 0.4 mm, respectively (i.e., the score increased with the low‐contrast detectability).

### E. Dynamic range

On the X‐ray display monitor, we also visually evaluated the dynamic range using both low and high attenuation (Cu thicknesses of 0.5 and 3.0 mm). The minimum thickness of the aluminum in which a hole could be detected was evaluated with the 0.5‐ and 3.0‐mm‐thick copper bases. The identifiable minimum thickness was scored visually from 1 to 7 for minimum thicknesses of 2.7, 2.3, 1.9, 1.5, 1.1, 0.7, and 0.3 mm with the 3.0‐mm‐thick copper base and 1.3, 1.1, 0.9, 0.7, 0.5,

0.3, and 0.1 mm with the 0.5‐mm‐thick copper base. The scores for both the 0.5‐mm‐thick and 3.0‐mm‐thick copper bases were added together. The score increased with the dynamic range.

#### E.1 Image quality evaluation

The cineradiography and fluoroscopy images (using static images) on the X‐ray display monitors were evaluated visually by two observers who were radiology specialists. The quality of the images was evaluated using the average scores assigned by the two specialists. The interobserver difference in the visual evaluation of the image quality was up to 10%.

#### E.2 Statistical analysis

The correlations (image quality scores vs. entrance surface dose, frame dose, or pulse dose) were analyzed using linear regression.

## III. RESULTS

### A. Radiation dose measurement

For fluoroscopy, the average entrance surface dose of the 18 X‐ray systems using the 20‐cm‐thick acrylic plate was 13.9 (range 2.1–28.2) mGy/min (Table 1).

The average pulse dose after correcting for the fluoroscopy pulse rate was 0.021 (range 0.005–0.042) mGy/pulse (Table 1). For fluoroscopy, the maximum surface and pulse doses exceeded the minimum dose by 13.4 and 8.4 times, respectively.

For cineradiography, the average entrance surface dose of the 18 X‐ray systems using the 20‐cm‐thick acrylic plate was 24.6 (range 5.1–49.3) mGy/10 s (Table 1). The average frame dose (after correcting for the cine frame rate) was 0.171 (range 0.042–0.329) mGy/frame (Table 1). For cineradiography, the maximum surface and frame doses exceeded the minimum dose by 9.7 and 8.25 times, respectively, using a 20‐cm‐thick acrylic plate.

### B. Image quality and radiation dose

#### B.1 Fluoroscopy

**Figure 2:**
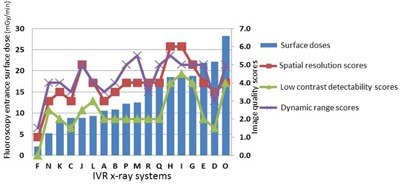
Fluoroscopy entrance surface doses of acrylic plates and image quality scores of 18 X‐ray systems (in entrance surface dose order). Entrance surface dose vs. image quality scores (spatial resolution, r=0.54; low‐contrast detectability, r=0.55; and dynamic range, r=0.49)

Figure 2 shows the relationship between the image quality scores and entrance surface dose. Low correlations were identified between the entrance surface dose and image quality scores (spatial resolution, r=0.54; low‐contrast detectability, r=0.55; and dynamic range, r=0.49).

**Figure 3:**
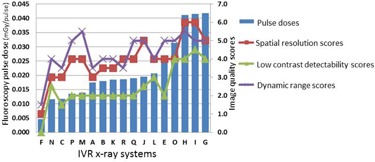
Fluoroscopy pulse doses and image quality scores of 18 X‐ray systems (in pulse dose order). Pulse dose vs. image quality scores (spatial resolution, r=0.85; low‐contrast detectability, r=0.92; and dynamic range, r=0.66).

Figure 3 shows the relationship between the image quality scores and pulse dose. There were significant positive correlations between the pulse dose and image quality scores (spatial resolution, r=0.85; low‐contrast detectability, r=0.92; and dynamic range, r=0.66).

**Figure 4:**
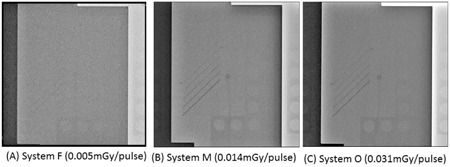
Fluoroscopy images of the QC phantom with the 1.5‐mm‐thick copper base: (a) very low, (b) intermediate, and (c) very high pulse doses. Note that (a) is much noisier because a very low dose was used.

Figure 4 shows examples of fluoroscopic images of the phantom taken using the minimum (System F), intermediate (System M), and very high (System O) pulse doses in FPD systems. The minimum pulse dose (System F) provided an obviously low‐quality image.

#### B.2 Cineradiography

**Figure 5:**
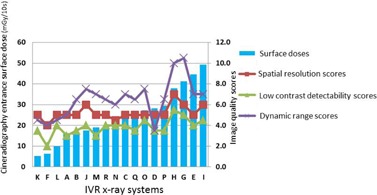
Cineradiography entrance surface doses and image quality scores in 18 X‐ray systems (in entrance surface dose order). Entrance surface dose vs. image quality scores (spatial resolution, r=0.56; low‐contrast detectability, r=0.65; and dynamic range, r=0.62)

Figure 5 shows the relationship between the image quality scores and entrance surface dose. There were positive correlations between the entrance surface dose and image quality scores (spatial resolution, r=0.56; low‐contrast detectability, r=0.65; and dynamic range, r=0.62). Figure 6 shows the relationship between the image quality scores and frame dose. There were similar positive correlations with the entrance surface dose between the frame dose and the image quality scores (spatial resolution, r=0.55; low‐contrast detectability, r=0.66; and dynamic range, r=0.57).

Examples of cineradiography images of the phantom taken using the minimum, intermediate, and maximum frame doses in FPD systems are shown in Fig. 7. The minimum frame dose FPD system (System F) had a tolerably low image quality. Furthermore, the images produced by the maximum frame dose (System I) and intermediate dose (System M) FPD systems were of similar quality.

**Figure 6:**
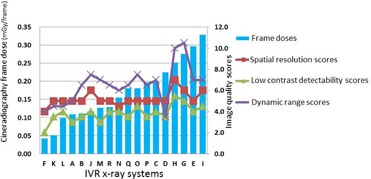
Cineradiography frame doses and image qualitv scores in IS X‐

**Figure 6:**
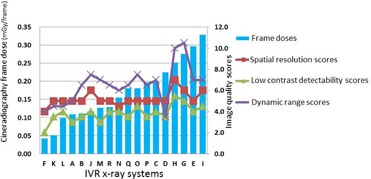
Cineradiography frame doses and image quality scores in 18 X‐ray systems (in frame dose order). Frame dose vs. image quality scores (spatial resolution, r=0.55; low‐contrast detectability, r=0.66; and dynamic range, r=0.57).

**Figure 7:**
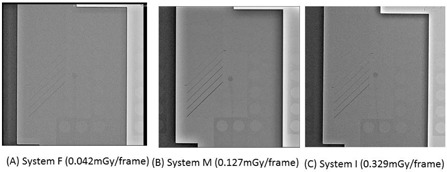
Cineradiography images of the QC phantom with the 1.5‐mm‐thick copper base: (a) very low, (b) intermediate, and (c) very high frame doses.

## IV. DISCUSSION

It is important to be aware of the relationship between image performance and radiation dose (i.e., increasing the dose improves the quality of the image, in general). Conversely, the tradeoff for a reduction in the radiation dose is a loss of image quality. The optimal input dose mode is that which achieves the best balance between image noise and radiation dose. With a very low dose, the diagnostic ability is obviously reduced. However, in the presence of some image noise, there may be no deterioration in the diagnostic ability. Equally, with a very high dose (very low image noise), the diagnostic ability is not necessarily improved compared with the diagnostic ability for a suitable radiation dose. In other words, the diagnostic ability may be similar for intermediate and very high radiation doses.

Naturally in our study, cine frame doses were higher than fluoroscopy pulse doses; hence, cine image quality scores were higher than fluoroscopy image quality scores.

One of the disadvantages associated with IVR is the dose of radiation received by patients and staff. Therefore, and especially in IVR, it is important to optimize the patient radiation dose and image performance. To our knowledge, this is the first detailed report to evaluate the radiation dose and image quality in multiple IVR systems. We found that the differences in radiation doses (fluoroscopy dose and pulse dose, cineradiography dose, and frame dose) among the 18 IVR X‐ray systems were very large. The differences in image quality scores (spatial resolution, low‐contrast detectability, and dynamic range) among the 18 IVR X‐ray systems were also very large.

Therefore, it may be necessary to optimize the radiation dose of IVR X‐ray systems according to image quality. In this study, we found positive correlations between radiation doses and image quality scores, in general, especially for fluoroscopy.

With fluoroscopy, System F resulted in very low radiation doses, but it also gave very low image quality scores. To increase the image quality, the image receptor input fluoroscopy dose for System F may need to be increased.

When the identifiable minimum diameter of the piano wire was 0.35 mm, the visual score (spatial resolution) was 3; 0.35 mm is the same diameter as the 0.014‐inch guidewire frequently used in PCI.

In fluoroscopy, it is thought that the visual score for the identifiable minimum diameter is greater than 3 in PCI. In our study, when the visual score was 3, the minimum dose of the fluoroscopy pulse dose was approximately 0.012 mGy/pulse. Therefore, the 0.012 mGy/pulse may become a criterion for the fluoroscopy pulse dose (entrance surface pulse dose) in PCI.

In cineradiography, the image quality of System I (maximum dose) was almost equivalent to that of System M (intermediate dose). Therefore, the image receptor input cineradiography dose for System I may need to be reduced, if there is no deterioration in diagnostic ability. Furthermore in cineradiography, there is a low correlation between radiation dose and image quality.

However, there may be a correlation between image quality scores and cineradiography pulse doses up to approximately 0.1 mGy/frame, and there may be a threshold dose (approximately 0.15 mGy/frame) — that is to say, image quality scores do not increase above the threshold dose (approximately 0.15 mGy/frame). Further detailed investigation will be required to clarify the explanation.

Furthermore, a phantom with vessels containing iodinated contrast may better relate to clinical imaging tasks than piano wires, especially the evaluation of cineradiography images.

The SDM used for dose measurement in the present study has a relatively low sensitivity. Therefore, there may be some errors in low‐dose measurements.

We routinely used a 20‐cm‐thick acrylic plate (a standard absorption object). When the thickness was changed to 15 or 25 cm, the X‐ray outputs (kV and the internal filters) were changed. Thus, both the radiation dose and image quality vary under the latter conditions.

Furthermore, when the field size (i.e., the field magnification mode of the image receptor) was changed, the radiation dose and image quality also varied.

To optimize radiation doses and image quality, periodical evaluations of radiation doses and image quality are required for both fluoroscopy and cineradiography IVR systems. Although periodic measurements are important, we did not seek to define an optimal test frequency in the present study.

This study used a static phantom. Further studies to evaluate the quality of moving images using a dynamic phantom may need to be undertaken.^28^, ^29^

## V. CONCLUSIONS

This paper described the relationship between radiation dose and image quality, in both fluoroscopy and cineradiography, based on 18 IVR X‐ray systems. The major findings of the article are that dose rates and image quality vary widely between the tested IVR systems. We found that a comparison of pulse and frame dose is more useful than dose rate (per minute or per second) in image quality evaluation. Furthermore, the 0.012 mGy/pulse may become a criterion for the fluoroscopy pulse dose (entrance surface pulse dose) in PCI.

In general, there tended to be a correlation between radiation dose and image quality. For pulse dose (fluoroscopy), there were significant correlations between dose and image quality (pulse dose vs. spatial resolution, r=0.85; pulse dose vs. low‐contrast detectability, r=0.92). The differences in surface dose among the 18 IVR X‐ray systems were high (max/min, 9.7‐fold for cineradiography; 13.4‐fold for fluoroscopy). Since some of the equipment results in high radiation doses, it will be necessary to consider reducing the radiation dose. The differences in image quality among the 18 cardiac IVR X‐ray systems were also high. In the IVR X‐ray systems with the lowest image quality, an increased radiation dose may be necessary. In fluoroscopy and cineradiography IVR X‐ray systems, it is important that both the radiation dose and image quality are managed and evaluated.

## ACKNOWLEDGMENTS

We thank Isao Yanagawa of the Tohoku University Hospital, Hideyo Azuma of the Sendai National Hospital, Yasushi Kudo of the Sendai City Hospital, Yoshihisa Kanno of the Tohoku Kosai Hospital, Yuji Kaga of the Sendai Kousei Hospital, Hiroo Chiba of the Tohoku Kouseinenkin Hospital, Taiki Chiba of the Sendai Open Hospital, Kunihiko Sato of the South Miyagi Medical Center, Yoshiharu Tada of the Tohoku Rosai Hospital, Hitoshi Numata of the Miyagi Shakaihoken Hospital, Takuya Yamashita of the Sendai Tokushukai Hospital, Satoru Toriyabe of the Sendai Shakaihoken Hospital, and Hisaya Shirotori of the Oosaki City Hospital, for their invaluable assistance.

This work was supported in part by a Grant‐in‐Aid for Scientific Research (24300179, 26460716) from the Japan Society for the Promotion of Science.

## COPYRIGHT

This work is licensed under a Creative Commons Attribution 3.0 Unported License.
